# A randomized comparison of a commercially available portion-controlled weight-loss intervention with a diabetes self-management education program

**DOI:** 10.1038/nutd.2013.3

**Published:** 2013-03-18

**Authors:** G D Foster, T A Wadden, C A LaGrotte, S S Vander Veur, L A Hesson, C J Homko, B J Maschak-Carey, N R Barbor, B Bailer, L Diewald, E Komaroff, S J Herring, M L Vetter

**Affiliations:** 1 Center for Obesity Research and Education, Temple University, Philadelphia, PA, USA; 2 Center for Weight and Eating Disorders, Perelman School of Medicine at the University at Pennsylvania, Philadelphia, PA, USA; 3 Division of Diabetes, Endocrinology and Metabolism, Department of Medicine, Temple University, Philadelphia, PA, USA; 4 Department of Public Health, Temple University, Philadelphia, PA, USA; 5 Division of Endocrinology, Diabetes, and Metabolism, Department of Medicine, Perelman School of Medicine at the University of Pennsylvania, Philadelphia, PA, USA

**Keywords:** portion control, diabetes, obesity, glycemic control, diabetes education

## Abstract

**Objective:**

This study examined the efficacy of a commercially available, portion-controlled diet (PCD) on body weight and HbA_1c_ over 6 months in obese patients with type 2 diabetes.

**Research Design and Methods:**

One-hundred participants with a mean±s.d. age of 55.6±10.6 year, body weight of 102.9±18.4 kg and HbA_1c_ of 7.7±1.3% were randomly assigned to a 9-session group lifestyle intervention that included a PCD or to a 9-session group program of diabetes self-management education (DSME). Participants in the two groups were prescribed the same goals for energy intake (1250–1550 kcal per day) and physical activity (200 min per week).

**Results:**

While both groups produced significant improvements in weight and HbA_1c_ after 6 months of treatment, PCD participants lost 7.3 kg [95% confidence interval (CI): −5.8 to −8.8 kg], compared with 2.2 kg (95% CI: −0.7 to −3.7 kg) in the DSME group (*P*<0.0001). Significantly more PCD than DSME participants lost ⩾5% of initial weight (54.0% vs 14.0%, *P*<0.0001) and ⩾10% (26.0% vs 6.0%, *P*<0.0001). HbA_1c_ declined by 0.7% (95% CI: −0.4 to −1.0%) in the PCD group, compared with 0.4% (95% CI: −0.1 to −0.7%) in DSME (*P*<0.026). Across both groups, larger weight losses were associated with greater reductions in HbA_1c_ (*r*=0.52, *P*<0.0001).

**Conclusions:**

These findings demonstrate that a commercially available portion-controlled meal plan can induce clinically meaningful improvements in weight and glycemic control in obese individuals with type 2 diabetes. These data have implications for the management of obesity in primary care, as now provided by the Centers for Medicare and Medicaid Services.

## Introduction

A 5–10% reduction in initial weight dramatically decreases the risk of developing type 2 diabetes in overweight persons with impaired glucose tolerance^1, 2^ and improves glycemic control in individuals who already have diabetes.^3, 4, 5^ Comprehensive programs of lifestyle modification reliably produce these improvements in weight (and glycemic control), but such interventions are often very intensive and limited to academic medical centers.^6, 7, 8^ Less intensive yet structured weight-loss programs, which incorporate lifestyle modification and portion-controlled meal replacement products potentially offer an important treatment option for overweight individuals with diabetes, as suggested by a recent study.^9^ Individuals who were provided weekly group lifestyle modification classes for 3 months and a diet of portion-controlled foods lost 7.1% of initial weight and reduced their hemoglobin A_1c_ (HbA_1c_) by 0.88%. Control participants, who received three standard diabetes education classes, achieved significantly smaller reductions of 0.4% in initial weight and 0.03% in HbA_1c_.^9^

The present 6-month randomized trial extends the prior study by standardizing the duration and intensity of the group treatment to isolate the effects of the portion-controlled diet (PCD) on the observed improvements in weight and glycemic control. To this end, overweight individuals with type 2 diabetes in each of the two treatment groups were prescribed the same calorie intake and physical activity goals and received the same number of group treatment sessions. Holding these variables constant across the two groups allowed for a clearer assessment of the effects of the PCD. We also elected in the present study to decrease the intensity (frequency) of the lifestyle intervention, a change that potentially could increase the dissemination of this approach.^10^

## Materials and Methods

### Participants

Participants were a total of 100 men and women who were recruited and treated at two medical centers in Philadelphia. The two sites followed identical protocols and met regularly to review study implementation. Participants were recruited from newspaper advertisements, flyers and physician referrals. Inclusion criteria included a body mass index of 25−50 kg m^−2^, age of 21−75 year, and a screening HbA_1c_ ⩾6.5% and <12.0%. Use of all classes of diabetes medications was permitted (including insulin), provided they had been prescribed at stable doses for ⩾3 months (or ⩾6 months in the case of incretin mimetics and pramlintide), as was the case for medications for other conditions (for example, hypertension, dyslipidemia). Exclusion criteria included significant cardiovascular, pulmonary, hepatic, renal or gastrointestinal disease; uncontrolled hypertension (⩾160/100 mm Hg); history of alcohol or drug abuse; significant psychiatric conditions thought to impair the candidate's safe or successful study participation; use of psychiatric medications (except for selective serotonin reuptake inhibitors); pregnancy or lactation; bariatric surgery; use of weight-loss inducing medications or supplements in the prior 3 months; or weight loss ⩾5 kg in prior 6 months. All applicants completed a history and physical examination, performed by their own primary care provider (PCP), who forwarded findings to the study physicians. (If the applicant's PCP was unavailable, the examination was completed by one of the study's nurse practitioners or physicians.) The study physicians provided final approval for applicants to participate.

**Figure 1:**
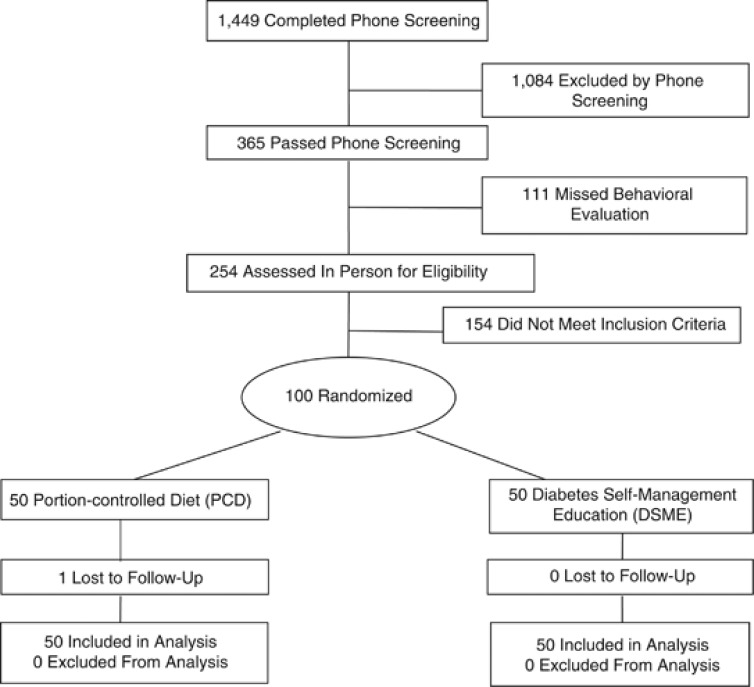
The flow of participants through the study.

All participants gave written informed consent to take part in the study, which was approved by the Institutional Review Boards at Temple University and the University of Pennsylvania. The first participant was randomized on 7 July 2010, and final outcome measures were obtained on 26 July 2011. Figure 1 shows the flow of participants through the study.

### Procedures

Participants were randomly assigned within site (stratified for insulin use), via a random-number generator, to: (1) a lifestyle intervention that included a prepackaged, PCD; or (2) a program of diabetes self-management education (DSME). The study statistician generated the random allocation sequence, and research coordinators enrolled participants and randomly assigned them to treatment conditions. The study's primary outcome was change in weight at 6 months. The principal secondary outcome was change in HbA_1c_ at 6 months.

### Interventions

Participants were randomized to one of two treatment conditions: Lifestyle intervention, PCD program or a DSME program.

#### Common elements

Participants in both treatment conditions attended group sessions (at weeks 0, 1, 2, 4, 8, 12, 16, 20 and 24) which lasted 90 min, included 8–12 persons, and were led by experienced practitioners (that is, lifestyle interventionists or certified diabetes educators, as appropriate). Participants in both treatment conditions were instructed to consume ∼1250 kcal per day (women) or 1550 kcal per day (men) and were provided a calorie-counting guide^11^ to facilitate this goal. Participants were instructed to gradually increase their walking (or other aerobic activity) to ⩾200 minutes per week.

All participants were instructed to monitor and record their blood glucose at least twice daily for 1 week before beginning their intervention. They were provided with a glucometer (OneTouch, LifeScan, Inc., Milpitas, CA, USA) and test strips for this purpose. Participants continued to monitor their blood glucose at least twice daily throughout the study, and records were reviewed by study staff at each session. If participants reported repetitive episodes of hyper- (three or more episodes ⩾300 mg dl^−1^) or hypoglycemia (three or more episodes ⩽60 mg dl^−1^), they received counseling regarding appropriate lifestyle strategies and medication adherence. Hypo- and hyperglycemia also prompted a referral back to PCP for medication adjustment. All changes in diabetic medications were managed by the participants' providers.

Participants in both treatment conditions met with a study physician at week 16 to review any changes in their health, including in blood pressure and serum chemistries (for example, triglycerides and total cholesterol) that were observed at the 3-month study assessment. Interventions differed from each other as described below.

#### Lifestyle intervention, PCD program

Half of the participants (*N*=50) were assigned to a lifestyle intervention that included the use of a prepackaged, PCD (Nutrisystem D, Fort Washington, PA, USA). Lifestyle intervention topics covered at group sessions included self-monitoring of food intake and physical activity, stimulus control, goal setting, problem solving, cognitive restructuring and relapse prevention.^6, 7^ This curriculum closely resembles that of the lifestyle modification guide included with the PCD program. Sessions began with a review of participants' progress from the prior meeting, followed by the introduction of a new topic in weight management.

Participants who received the PCD were instructed to supplement the prepackaged foods (which typically provided three entrées and one snack daily) with conventional foods (for example, fruits, vegetables, dairy items and lean protein), in accordance with the PCD program. Women's and men's meal plans provided ∼1250 and 1550 kcal per day, respectively, with ∼55% of total energy from the packaged foods and 45% from supplemental grocery items. The plans were structured to provide ∼50–55% of energy from carbohydrate, 20–25% from fat and 20–25% from protein, and to contain ⩽2300 mg per day of sodium. The glycemic index of the diet (including both the packaged and conventional foods) was ∼34 (on the glucose scale), which falls in the low range.^12, 13^

#### DSME program

The remaining participants (*N*=50) received a multi-faceted diabetes education program, which addressed topics that included goal setting, nutrition, weight loss, blood glucose monitoring, physical activity, prevention and management of hyperglycemia and hypoglycemia, sick day management, prevention of complications, medication management and barriers, and coping with diabetes. The program was based on the American Diabetes Association's National Standards for DSME.^14^ At the initial session each participant selected a specific goal. Sessions, led by a certified diabetes educator, were conducted in a format similar to the PCD group, beginning with a review of the prior session's readings and homework and followed by the introduction of a new topic. The DSME was intended to model structured diabetes self-management training programs provided by certified diabetes educators in medical settings.

Participants were instructed to consume a balanced deficit diet consistent with recommendations of the Food Guide Pyramid^15^ and the American Diabetes Association.^16^ The suggested macronutrient content was 15–25% of calories from protein, <30% from fat (<7% from saturated fat) and the remainder from carbohydrate, with 30 g per day of fiber. Meal plans were provided to help participants meet their nutrition and calorie goals.

### Outcomes

The following outcomes were assessed at baseline and 3 and 6 months.

#### Weight and height

Weight was measured on a calibrated electronic scale (Detecto 758C Digital weight indicator, Webb City, MO, USA), with participants dressed in light clothing, without shoes. Height was measured at baseline using a wall-mounted stadiometer (Holtain Limited Harpenden Stadiometer, Crymych Dyfed, UK). Body mass index was calculated as weight (kg) divided by height (m)^2^.

#### Waist circumference

Waist circumference was measured in centimeters using a standard tape measure (Gulick II, Country Technology, Gays Mills, WI, USA). Participants remained standing while the tape was placed around the abdomen horizontally at the midpoint between the highest point of the iliac crest and the lowest part of the costal margin. Waist circumference was measured three times, and the average of the three readings was used.

#### Blood pressure

Blood pressure was assessed using an automated instrument (Dinamap ProCare 200, GE Medical Systems, Milwaukee, WI, USA) with cuff sizes based on measured arm circumference. After sitting quietly for 5 min, two readings were taken, separated by a 1-minute rest period. The average of the two readings was used.

#### Serum chemistries

HbA_1c_, glucose, hs-CRP and lipid values were measured from samples obtained after participants fasted overnight (12 h). Samples, other than HbA_1c_, were centrifuged to separate serum and were shipped overnight to a commercial lab where the assays were performed (Quest Diagnostics, Horsham, PA, USA). (Details of each assay are described at http://www.questdiagnostics.com/hcp/qtim/testMenuSearch.do.) HbA_1c_ was assayed at Temple University using high-performance liquid chromatography.

#### Diabetes medication regimen

At baseline, participants reported all medications that they took for diabetes. The name, dosage, frequency, and start date of each drug was recorded. Throughout the trial, changes in diabetes medication use were recorded in the study medical record. At 3 and 6 months, a reduction in the intensity of the diabetes medication regimen was operationalized as taking fewer medications or a lower dosage, compared with baseline. By contrast, an increase was defined as the addition of new medication or a higher dosage, compared with the baseline regimen. When a participant had changes in both directions (for example, discontinued one medication and started another), the net change in treatment intensity was adjudicated by a study physician who was masked to the patient's identity or treatment condition.

#### Statistical analyses

Differences between the groups on baseline characteristics were assessed using independent samples *t*-tests for continuous variables and chi square *χ*^2^-tests for categorical variables. The primary outcome was the change in body weight (kg) at month 6, as determined using a linear mixed-effects model with time, treatment and a time by treatment interaction included as explanatory variables. This model posited an unrestricted structure on the variance-covariance matrix of the residuals on all 100 participants. All continuous secondary outcomes were analyzed in the same manner as body weight. Categorical secondary outcomes (for example, changes in medication regimen intensity, achievement of clinically meaningful targets for weight loss and HbA_1c_) were analyzed using *χ*^2^-tests or Fisher's Exact Test. Alpha was set at *P*<0.05 for the comparison of the two treatment conditions on the primary outcome (weight change), as well as on all secondary outcomes. All statistical analyses were performed with SAS 9.2 (SAS Institute Inc., Cary, NC, USA).

## Results

### Participants' baseline characteristics

Study participants were 59 women and 41 men with a mean±s.d. age of 55.6±10.6 years, weight of 102.9±18.4 kg, body mass index of 35.8±5.3 kg m^−2^, and HbA_1c_ of 7.7±1.3%. The sample included 59 African Americans, 36 Caucasians and 3 Asian Americans, as ascertained by self-report. The two treatment conditions did not differ significantly on any baseline characteristics (as shown in Table 1). As shown in Figure 1, 49 of 50 participants in PCD completed the 6-month outcome, as did 50 of 50 in DSME.

### Weight loss

**Figure 2:**
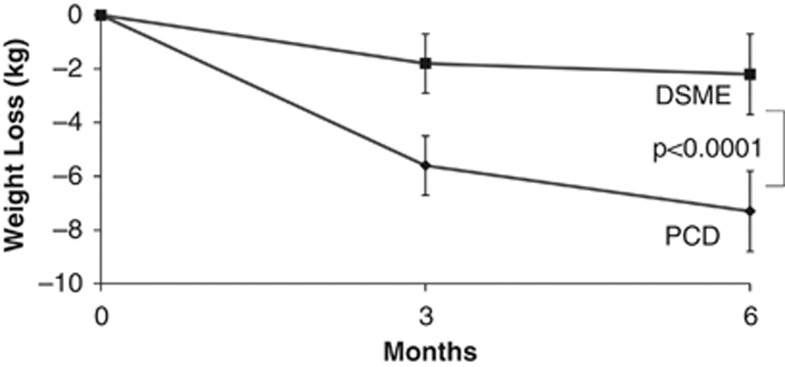
The figure shows weight loss (in kg) in participants assigned to the PCD and DSME conditions.

At month 6, PCD participants lost 7.3 kg (95% confidence interval (CI): −5.8 to −8.8 kg), compared with a significantly (*P*<0.0001) smaller 2.2 kg loss (95% CI: −0.7 to −3.7 kg) for DSME (see Table 2 and Figure 2). These losses corresponded to reductions in initial weight of 7.8% and 2.1% for PCD and DSME, respectively. Figure 2 shows that directionally similar differences were observed between the groups at month 3 (−5.6 vs −1.8 kg). A significantly greater percentage of participants in the PCD than DSME groups lost ⩾5% of initial weight at month 6 (54.0% vs 14.0%, *P*<0.0001), as well as ⩾10% (26.0% vs 6.0%, *P*<0.0001) (note: the percentage of participants who lost ⩾5% includes those who lost ⩾10%).

### Change in HbA_1c_

At month 6, HbA_1c_ declined by 0.7% (95% CI: −0.4 to −1.0%) in PCD participants, compared with a significantly (*P*<0.026) smaller 0.4% decline (95% CI: −0.1 to −0.7%) in DSME participants (see Table 2). Similar reductions were observed in the two groups at month 3 (−0.8% vs −0.4%). At month 6, a significantly (*P*<0.0046) greater percentage of participants in the PCD than DSME groups (72.0 vs 44.0%) met the American Diabetes Association goal of tight blood sugar control (that is, HbA_1c_<7.0%).^17^ Corresponding values at baseline had been 38.0% and 36.0%, respectively.

### Change in use of diabetes medications

At baseline, 94.0% of participants in both the PCD and DSME groups were taking an oral agent for their diabetes, the most common of which was metformin, used by 82% and 86% of participants in the two groups, respectively. Insulin was used by 18% and 22% of participants, respectively, and non-insulin injectables by 8% and 0% of individuals, respectively. At month 6, 28.0% of PCD participants, compared with 4.0% of DSME, had a reduction in the intensity of their diabetes medication regimen (*P*=0.0034). By contrast, 6.0% of PCD vs 12.0% of DSME participants had an intensification in their regimen.

### Additional secondary outcomes

As shown in Table 2, participants in both conditions achieved significant reductions from baseline in body mass index, waist circumference, triglycerides and total cholesterol, with PCD participants achieving significantly greater reductions than DSME participants in waist circumference (*P*<0.0001) and in systolic blood pressure (*P*=0.044) at month 6. The former group generally achieved more favorable changes than the latter on additional measures of cardiovascular disease risk. However, differences between the groups were not statistically significant.

### Relationship between weight change and HbA_1c_change

The two treatment conditions were combined (*N*=100) to examine the relationship between changes in weight and HbA_1c_ at month 6. Partial correlation analysis, which controlled for the effect of treatment condition, revealed that the more weight participants lost (kg), the greater their reduction in HbA_1c_ (*r*=0.52, *P*<0.0001).

### Adverse events

Two participants, both in the PCD group, experienced serious adverse events. The first had urinary retention and hematuria (at week 18). The second reported (at week 20) that he had experienced a myocardial infarction between his screening and baseline visits. (This information was withheld from study staff until week 20.) This individual also was hospitalized for atrial fibrillation at week 25 (1 week after the study ended; reported to study staff at week 28). None of the serious adverse events was determined to be related to study treatment.

## Discussion

This study's principal finding was that a lifestyle intervention that incorporated a PCD of prepackaged foods produced a 7.3 kg weight loss in 6 months that was associated with a clinically significant reduction in HbA_1c_ of 0.7% and with reductions in the use of diabetes medications. Improvements on all three measures were superior to those produced by a robust control intervention of DSME that also was associated with significant reductions in weight and HbA_1c_. A meta-analysis of diabetes self-management programs^18^ found a 0.73% decrease in HbA_1c._ It is important to note, however, that only 4 of the 29 studies had baseline HbA_1c_ comparable to our study (that is,<8). In addition, DSME is typically applied in either newly diagnosed patients or to those experiencing complications. Thus, the higher initial HbA_1c_ values in that meta-analysis likely led to greater reductions than were observed in our DSME group.

The low glycemic index (GI) of the PCD may have added to the effect of weight loss on HbA1c. A review of relatively small and short studies found significantly greater reductions in glycated proteins with lower-GI diets than higher-GI alternatives.^19^ More recently, Fabricatore *et al.*^13^ found a significantly greater reduction in HbA1c with a low-glycemic load diet (also low in GI) compared with an isoenergetic low-fat diet, despite equivalent weight loss, in a sample of obese adults with type 2 diabetes. Although we do not know whether or to what extent dietary GI differed between the two groups in the present study, the very low GI—34—of the PCD makes it reasonable to assume that the diet consumed in the DSME group was considerably higher in GI.

This study demonstrates the benefits of prescribing a low-calorie diet that incorporates prepared portion-controlled foods. At month 6, participants who were provided the PCD plan lost 5.1 kg more than individuals who were instructed to consume an equivalent-calorie diet of self-selected foods; both dietary interventions were consistent with recommendations of the American Diabetes Association.^16^ The present findings confirm that low-calorie portion-controlled foods, whether provided as liquid shakes and meal bars,^20, 21^ or as prepared servings of conventional foods,^22^ induce significantly greater weight losses than recommendations to consume an equivalent-calorie diet comprised entirely of self-selected foods. This finding has been observed in overweight and obese individuals with^22, 23, 24^ and without^20, 25, 26^ type 2 diabetes. PCDs help those trying to manage their weights meet their calorie goals by providing pre-measured amounts of food with a known energy intake.^21, 27^ By contrast, overweight and obese individuals typically underestimate their energy intake by 40% or more when consuming a self-selected diet of conventional foods.^28^

Participants in the PCD group achieved a 7 kg weight loss and clinically significant metabolic improvements with only nine group treatment sessions over 24 weeks. In most lifestyle modification trials, investigators typically have prescribed weekly counseling sessions for the first 16 to 26 weeks^6, 7^ or, as in the case of the Diabetes Prevention Program, 16 sessions over 26 weeks.^1^ The inclusion of a PCD would appear to allow participants to achieve clinically meaningful weight loss in fewer sessions than required if a traditional low-calorie diet is used. Fewer treatment sessions could be more convenient to patients, as well as reduce the cost of achieving clinically significant weight loss. The Centers for Medicare and Medicaid Services, for example, have agreed to reimburse the cost of lifestyle counseling for weight control.^29^ Patients will be provided 14 to 15 visits with a PCP over 6 months, with the goal of losing at least 3 kg. Inclusion of a PCD with lifestyle counseling would appear likely to increase the magnitude of the weight loss and, with it, improvements in health. A more rapid rate of weight loss also could possibly reduce the number of PCP visits required, thus reducing costs. The weight reduction achieved in the DSME group (2.2 kg with 9 sessions over 6 months) suggests the potential that diabetes educators who prescribed energy restriction through prepackaged meals would also induce a mean weight loss that exceeds the Centers for Medicare and Medicaid Service goal, if provided 14 sessions in which to deliver the intervention. Such an approach may be a suitable, lower-cost alternative to physician-delivered intensive behavioral counseling for obese individuals with type 2 diabetes.

Overweight and obese individuals typically are advised to lose 5–10% of their initial weight to improve health complications.^3, 30^ The present findings support this recommendation and demonstrate the benefits of achieving larger weight loss, ∼10% of initial weight rather than 5%. Collapsing across the treatment groups we observed a strong, linear relationship between reductions in weight and HbA_1c._ The use of the PCD in the present study significantly increased the number of participants who lost 10% or more of weight, with the accompanying benefit of the reduction in HbA_1c_.

In addition to its strengths, this study also had some limitations, the principal of which was the absence of a follow-up assessment. We anticipate that participants would require continued intervention, as provided in the Look AHEAD study,^5, 31^ to maintain their full improvements in weight and HbA_1c_. The study also was underpowered to assess clinically significant differences in secondary outcomes. Moreover, 81% and 63% of participants in the total sample took medications for hypertension and dyslipidemia, respectively (data not shown), which helps to explain the relatively low baseline values we observed for these cardiovascular disease risk factors. A third limitation was the lack of a usual care control group. Given that fewer than 60% of adults with diabetes reported ever having attended a diabetes self-management class,^32^ both study conditions offered a stronger behavioral intervention than what most patients with type 2 diabetes are likely to receive in practice. Finally, the lack of self-reported data on physical activity and diet precluded an assessment of adherence to the prescribed regimens.

## Conclusion

In summary, this study found that a lifestyle intervention that included a PCD induced significantly greater weight loss and improvements in HbA_1c_ in 6 months than did a comprehensive diabetes self-management and education program that provided instruction in weight management and control of diabetes complications. These findings suggest that traditional diabetes education programs, as offered by diabetes educators (as in the present study), could be strengthened by the addition of a PCD.

## Figures and Tables

**Table 1:** Baseline characteristics of participants

*Measures*	*PCD*	*DSME*	*All*
*N*	50	50	100
*Gender, no. of participants (%)*			
Male	21 (42.0)	20 (40.0)	41 (41.0)
Female	29 (58.0)	30 (60.0)	59 (59.0)
			
*Race, no. of participants (%)*			
White	16 (32.0)	20 (40.0)	36 (36.0)
African-American	32 (64.0)	27 (54.0)	59 (59.0)
Asian	2 (4.0)	1 (2.0)	3 (3.0)
Hispanic/Latino	1 (2.0)	1 (2.0)	2 (2.0)
Other/more than on race	0 (0.0)	2 (4.0)	2 (2.0)
			
Age (year)	55.5±10.3	55.7±11.0	55.6±10.6
Body mass index (kg m^−2^)	35.3±4.6	36.2±5.8	35.8±5.3
Weight (kg)	101.8±16.7	104.0±20.1	102.9±18.4
Waist circumference (cm)	115.9±12.1	118.4±14.8	117.1±13.5
Systolic blood pressure (mm Hg)	128.2±16.6	125.7±17.3	126.9±16.9
Diastolic blood pressure (mm Hg)	74.4±9.5	75.4±10.4	74.9±9.9
Hemoglobin A_1C_ (%)	7.6±1.3	7.9±1.3	7.7 ±1.3
Glucose (mg dl^−1^)	144.2±44.9	161.5±59.9	152.9±53.4
Total cholesterol (mg dl^−1^)	172.3±39.8	173.1±38.0	172.7±38.8
Triglycerides (mg dl^−1^)	136.5±60.7	141.0±78.5	138.8±69.9
High-density lipoprotein (mg dl^−1^)	49.9±16.8	51.8±15.3	50.8±16.0
Low-density lipoprotein (mg dl^−1^)	96.0±36.4	94.3^a^±30.5	95.1±33.5
hs-CRP (mg l^−1^)	5.6^a^±7.4	5.8±6.0	5.7±6.7

Abbreviations: All, both groups combined; DSME, diabetes self-management education program; hs-CRP, high-sensitivity CRP; PCD, portion-controlled diet.

Data are means±or frequency (%). There were no significant differences between the two groups.

a *N*=49.

**Table 2:** Change in primary and secondary outcomes at month 6

*Measure*	*PCD*	*DSME*	P*-value*
*N*, baseline (6 months)	50 (49)	50 (50)	
			
*Weight (kg)*			
Baseline	101.8±16.7	104.0±20.1	
6 Months	93.9±14.7	101.8±19.4	
Adjusted change	−7.3 (−8.8 to −5.8)	−2.2 (−3.7 to −0.7)	<0.0001
			
Body mass index *(kg m*^−*2*^)			
Baseline	35.3±4.6	36.2±5.8	
6 Months	32.6±4.2	35.5±5.8	
Adjusted change	−2.5 (−3.0 to −2.0)	−0.7 (−1.2 to −0.2)	<0.0001
			
*Waist circumference (cm)*			
Baseline	115.9±12.1	118.4±14.8	
6 Months	108.9±11.1	115.5±14.9	
Adjusted change	−6.5 (−7.8 to −5.3)	−2.9 (−4.1 to −1.7)	<0.0001
			
*Hemoglobin A*_*1C*_*(%)*			
Baseline	7.6±1.3	7.9±1.3	
6 Months	6.9±1.2	7.5±1.3	
Adjusted change	−0.7 (−1.0 to −0.4)	−0.4 (−0.7 to −0.1)	0.021
			
*Fasting glucose (mg dl*^−*1*^)			
Baseline	144.2±44.9	161.5±59.9	
6 Months	128.0±47.9	148.7±52.3	
Adjusted change	−16.5 (−33.0 to +0.1)	−12.8 (−29.3 to +3.7)	0.217^a^
			
*Systolic blood pressure (mm Hg)*			
Baseline	128.2±16.6	125.7±17.3	
6 Months	121.4±16.2	123.8±13.8	
Adjusted change	−6.6 (−11.0 to −2.2)	−1.9 (−6.2 to +2.5)	0.044^a^
			
*Diastolic blood pressure (mm Hg)*			
Baseline	74.4±9.5	75.4±10.4	
6 Months	72.4±11.2	74.1±9.8	
Adjusted change	−2.2 (−4.6 to +3.0)	−1.2 (−3.6 to +1.2)	0.359
			
*Triglycerides (mg dl*^−*1*^)			
Baseline	136.5±60.7	141.0±78.5	
6 Months	109.2±64.6	120.7±54.6	
Adjusted change	−26.3 (−42.4 to −10.3)	−20.3 (−36.3 to −4.3)	0.343^a^
			
*Total cholesterol (mg dl*^−*1*^)			
Baseline	172.3±39.8	173.1±38.0	
6 Months	162.5±42.2	165.3±29.6	
Adjusted change	−8.6 (−15.9 to −1.2)	−7.8 (−15.1 to −0.5)	0.808
			
*High-density lipoprotein (mg dl*^−*1*^)			
Baseline	49.9±16.8	51.8±15.3	
6 Months	50.1±14.8	50.2±13.8	
Adjusted change	−0.1 (−1.9 to +1.7)	−1.6 (−3.4 to +0.2)	0.113
			
*Low-density lipoprotein (mg dl*^−*1*^)			
Baseline	96.0±36.4	94.3±30.5^b^	
6 Months	90.3±36.1	93.0±26.6	
Adjusted change	−4.3 (−11.0 to +2.4)	−0.8 (−7.6 to +5.9)	0.320
			
*hs-CRP (mg l*^−*1*^)			
Baseline	5.6±7.4^b^	5.8±6.0	
6 Months	4.4±5.8	4.7±4.8	
Adjusted change	−1.0 (−2.5 to +0.5)	−1.2 (−2.6 to 0.3)	0.883

Abbreviations: DSME, diabetes self-management education program; hs-CRP, high-sensitivity CRP; PCD, portion-controlled diet

a Indicates that *P*-values were obtained from a linear mixed-effects model on the log-transformed outcome, which were similar in direction and significance to analyses performed on the raw outcome.

b Data were obtained for only 49 of 50 participants.

Unadjusted baseline and 6-month data are reported as means±s.d.

Adjusted change is reported as means (95% confidence intervals) and was obtained from linear mixed-effects models with time, treatment, and a time by treatment interaction included as explanatory variables.

## Notes

Dr Foster is a consultant with Nutrisystem. Dr Wadden is a consultant with BMIQ. All other authors declare no conflict of interest.
